# Low-cost exercise interventions improve long-term cardiometabolic health independently of a family history of type 2 diabetes: a randomized parallel group trial

**DOI:** 10.1136/bmjdrc-2020-001377

**Published:** 2020-11-20

**Authors:** Niko S Wasenius, Bo A Isomaa, Bjarne Östman, Johan Söderström, Björn Forsén, Kaj Lahti, Liisa Hakaste, Johan G Eriksson, Leif Groop, Ola Hansson, Tiinamaija Tuomi

**Affiliations:** 1 Department of General Practice and Primary Health Care, University of Helsinki, Helsinki, Finland; 2 Folkhälsan Research Center, Helsinki, Finland; 3 The Department of Social Services and Health Care, City of Jakobstad, Jakobstad, Finland; 4 Center of Excellence in Complex Disease Genetics, Institute of Molecular Medicine Finland, University of Helsinki, Helsinki, Finland; 5 Närpes Health Care Center, Närpes, Finland; 6 Vaasa Central Hospital, Vaasa, Finland; 7 Lund University Diabetes Centre, Depratment of Clinical Sciences, Lund University, Malmö, Sweden; 8 Department of Endocrinology, Abdominal Centre, Helsinki University Hospital, Helsinki, Finland; 9 Research Program Unit, Clinical and Molecular Metabolism, University of Helsinki, Helsinki, Finland

**Keywords:** cardiorespiratory fitness, diabetes mellitus, type 2, exercise, metabolism

## Abstract

**Introduction:**

To investigate the effect of an exercise prescription and a 1-year supervised exercise intervention, and the modifying effect of the family history of type 2 diabetes (FH), on long-term cardiometabolic health.

**Research design and methods:**

For this prospective randomized trial, we recruited non-diabetic participants with poor fitness (n=1072, 30–70 years). Participants were randomly assigned with stratification for FH either in the exercise prescription group (PG, n=144) or the supervised exercise group (EG, n=146) group and compared with a matched control group from the same population study (CON, n=782). The PG and EG received exercise prescriptions. In addition, the EG attended supervised exercise sessions two times a week for 60 min for 12 months. Cardiometabolic risk factors were measured at baseline, 1 year, 5 years, and 6 years. The CON group received no intervention and was measured at baseline and 6 years.

**Results:**

The EG reduced their body weight, waist circumference, diastolic blood pressure, and low-density lipoprotein-cholesterol (LDL-C) but not physical fitness (p=0.074) or insulin or glucose regulation (p>0.1) compared with the PG at 1 year and 5 years (p≤0.011). The observed differences were attenuated at 6 years; however, participants in the both intervention groups significantly improved their blood pressure, high-density lipoprotein-cholesterol, and insulin sensitivity compared with the population controls (p≤0.003). FH modified LDL-C and waist circumference responses to exercise at 1 year and 5 years.

**Conclusions:**

Low-cost physical activity programs have long-term beneficial effects on cardiometabolic health regardless of the FH of diabetes. Given the feasibility and low cost of these programs, they should be advocated to promote cardiometabolic health.

**Trial registration number:**

ClinicalTrials.gov identifier NCT02131701.

**Significance of this study**

**What is already known about this subject?**

- Family history of type 2 diabetes is associated with reduced physical fitness and a lower expression of genes in the oxidative phosphorylation.
- Limited prospective data are available on how type 2 diabetes or family history of type 2 diabetes affects physical fitness or metabolic response to exercise.

**What are the new findings?**

- Family history of type 2 diabetes attenuated the effect of exercise on waist circumference and low-density lipoprotein.
- One-year low-cost structured exercise intervention and exercise prescription intervention similarly led to an improved cardiometabolic health 6 years later compared with the population-based control group.

**How might these results change the focus of research or clinical practice?**

- Individuals with family history of type 2 diabetes can in most parts expect similar cardiometabolic health benefits from regular exercise; however, the diminished responsiveness to waist circumference and low-density lipoprotein warrants further investigation.
- Physical activity prescription could be a feasible and readily available option to promote long-term cardiometabolic health in low fit populations.

## Introduction

Regular physical activity has been associated with a myriad of health benefits including reduced risk of mortality, diabetes and other non-communicable diseases.^1–5^ Exercise interventions can also positively affect cardiometabolic health by improving maximum oxygen uptake (VO_2max_), insulin sensitivity, adiposity, LDL-cholesterol (LDL-C), and blood pressure.^6–8^

Although physical activity has a central role in the prevention and treatment of type 2 diabetes, the interindividual variability in response to exercise is large.^9 10^ Heterogeneity in VO_2max_ training response is strongly explained by heritability (47%), but only minimally by age, sex, and ethnic origin.^10 11^ A genetic component is supported by reports of reduced physical fitness and a lower expression of genes in the oxidative phosphorylation pathway in patients with type 2 diabetes and their family members.^12–15^ Family history of type 2 diabetes (FH) could also limit muscle adaptation to exercise and possible health benefits.^16^ However, only few prospective studies have investigated the influence of type 2 diabetes or FH on the metabolic response to exercise.^16 17^

Therefore, this study aimed to investigate the long-term (5 years) effect of exercise prescription versus a 1-year supervised exercise intervention (EG) on aerobic physical fitness, muscle strength, and cardiometabolic parameters in subjects with poor physical fitness, stratified for FH. We also compared the change in measured outcomes between the intervention groups and matched control subjects originating from the same population study at 6 years.

## Research design and methods

### Study participants

The Prevalence, Prediction and Prevention of diabetes (PPP)-Botnia Study is a population-based study (n=5 208) in Western Finland initiated to obtain accurate estimates of prevalence and risk factors for diabetes, pre-diabetes and the metabolic syndrome in the adult population and to use this information for prediction and prevention of the disease.^12^ The baseline study was conducted in 2004–2008 and the prospective study in 2010–2015.^18^ For the present randomized parallel group clinical trial (RCT, ClinicalTrials.gov identifier NCT02131701) we recruited consecutive male and female participants from the PPP-Botnia Study who fulfilled the following criteria: (1) no diabetes and aged 30–70 years; (2) poor physical fitness based on a 2 km walking test (fitness index <90); and (3) no contraindications for physical training based on a physical examination including ECG. Of the 507 consecutive eligible participants invited, 290 gave written informed consent. Included participants were randomized (1:1) in four blocks to an exercise group (EG) (n=146) and a prescription group (PG) (n=144) based on gender and FH, which was defined as one first-degree or two second-degree relatives with type 2 diabetes. The basal visit took place between October 2006 and August 2008. The 1-year follow-up study was performed in 2007–2009, and the 5-year follow-up was performed between 2011 and 2013. Participants or care providers were not blinded, as this is rather impossible. However, those assessing outcomes were blinded for allocation. All participants gave written informed consent before taking part to the study.

In addition to the exercise intervention groups, a population-based control group (CON; n=782) was recruited from the participants in the PPP-Botnia Study. The CON group received no specific intervention. Male and female participants were considered eligible for the CON group if they were (1) non-diabetic and aged 30–70 years, (2) had poor physical fitness based on a 2 km walking test (fitness index <90 in two walking tests) and (3) participated in both the baseline and 6-year follow-up study. The CON group was subdivided according to the FH by same criteria as the intervention groups. The baseline visits occurred 2004–2008 and 6-year follow-up visits 2010–2015. The online supplemental figure S1 describes the flow of the study.

10.1136/bmjdrc-2020-001377.supp1 Supplementary data

### Interventions

All study subjects participated in two individual sessions of exercise prescription aiming at 30 min of moderate exercise at least 5 days a week. They also received exercise diaries. Participants in the PG performed unsupervised training, while participants in the EG were offered supervised physical training in groups twice a week during 12 months. The supervised training included both endurance training (Nordic walking and water gymnastics) and resistance training in a gym. The training sessions lasted 60 min including warm-up. To control the intensity of aerobic training, 70%–85% of age-adjusted maximal pulse rate was recommended, and heart rate monitoring was used periodically. Muscle strength training was performed in two sets of 12–15 repetitions focusing on major muscle groups. The first set (warm-up) was performed with 10 repetitions at 50% of repetition maximum, and the second set at 70%. Resistance was progressively increased according to the repeated testing of muscle strength. Experienced trainer supervised the training and also performed the testing of the muscle strength.

### Timing of the measurements

In the PG and EG, all measurements were taken at baseline, 1 year, and 5 years. All measurements were also collected from the CON group at baseline. At 6-year follow-up, body composition, waist circumference, blood pressure, blood lipid and glucose metabolism were measured from all groups (PG, EG, and CON).

### Assessment of aerobic physical fitness, steps, and muscle strength

Aerobic physical fitness as a primary outcome was assessed in all subjects by a 2 km walking test (UKK), which provides an indirect estimate of oxygen uptake.^19 20^ Based on walking time and heart rate at the end of the test, a fitness index (<70: very poor, 70–89: poor, 90–110: normal, 111–130: good, and >130: very good) adjusted for age, gender and body mass index (BMI) was calculated. The repeated tests were performed on the same track and at the same time of the year. At baseline, the number of steps per day was measured with Actiheart 4 (CamNtech Ltd. UK).

Muscle strength (arm push, arm pull, leg extension and leg flexion) was assessed by fitness equipment (Ab Hur Oy, Finland) after a 5–10 min warm-up. Each subject performed five repetitions during 10 s. The resistance was increased until the subjects were unable to perform more. One repetition maximum (1 RM) was calculated based on the Brzyckis formula: 1 RM = weight/(1.0278 – (0.0278 × repetitions)).^21^ Assessment of muscle strength was carried out on all participants in the training group and in a subgroup of consecutive participants in the prescription-of-exercise group. Number of subjects who participated in muscle strength testing at baseline was 135 subjects (EG: n=106, PG: n=29), at 1 year 128 subjects (n=100 and n=28, respectively) and at 5 years 136 subjects (n=108 and n=28, respectively).

### Anthropometrics and blood pressure

Body weight (Tanita BF-350, Japan) and height were measured with subjects in light clothing without shoes, and BMI was calculated. Waist circumference was measured with a soft tape on standing subjects midway between the lowest rib and the iliac crest. Two blood pressure recordings (Omron 711) were obtained from the right arm of a sitting person after 30 min of rest at 5 min intervals, and their mean value was calculated.

### Questionnaires and diaries

Questionnaires were used to obtain information about other diseases, current medication, smoking and alcohol consumption. At baseline, 1 year and 5 year follow-up frequency and intensity of physical activity during the past 12 months was assessed using the validated Kuopio Ischemic Heart Disease questionnaire (KIHD).^22^ It provides detailed information on common lifestyle, commuting and leisure time physical activity and enables assessment of physical activity as metabolic equivalent of task (MET) hours per week (MET-hour/week). Furthermore, during the 1-year intervention, all participants in the PG and EG kept an exercise diary, in which they were instructed to log every exercise that they performed during the 1-year period. The activities were then transformed to MET-hours per week and categorized into aerobic, gym or resistance training type of exercises and other miscellaneous physical activities. Miscellaneous physical activities included non-exercise type of activities, for example, home activities, home repair, lawn moving and gardening activities. In addition to the exercise diary, participants in the EG were instructed to log every supervised exercise session, including type, duration, and frequency. They performed three types of exercise (resistance training, Nordic walking, or aquatic training). The physical dose (duration, frequency, intensity, and volume) of the training was calculated. For each type of supervised exercise, a MET-value was calculated based on the Compendium of Physical Activities.^23^ The time-weighted average intensity of the training was expressed in MET-values and volume in MET-hours.^24^ Information on possible adverse effects was collected based on the participants reporting during the intervention. A questionnaire was also used collect data on type blood pressure, cholesterol, and diabetes medication at baseline and at 6 years. The information of each medication was dichtomized (0=no and 1=yes).

### Analytical measurements

The subjects participated in an oral glucose tolerance test (OGTT) by ingesting 75 g of glucose (Glucodyn, Leiras, Turku, Finland) after a 12-hour overnight fast. Samples for the measurement of plasma glucose and serum insulin were drawn at 0, 30, and 120 min. Glucose and insulin area under curve (AUC glucose; AUC insulin=15 × fasting concentration+60 × concentration at 30 min+45 × concentration at 120 min) were also calculated. Fasting samples were drawn for the measurement of serum total cholesterol, high-density lipoprotein cholesterol (HDL-C), and triglyceride concentrations. LDL-C concentrations were calculated using the Friedewald formula.^25^

Serum insulin concentrations were measured with a fluorometric immunoassay (AutoDELFIA B080-101-assay, PerkinElmer, USA), plasma glucose by HemoCue Glucose 201-assay (HemoCue, Sweden), and serum lipids by an immunoturbidometric method (Konelab 60i 981700, Thermo Fisher Scientific, USA). Insulin sensitivity was assessed with homeostasis model assessment for insulin resistance (HOMA-IR=[(fasting serum insulin concentration/fasting plasma glucose concentration)/22.5]^26^ and with insulin sensitivity index (ISI=10 000/√(fasting glucose × fasting insulin × mean OGTT glucose × mean OGTT insulin)). As indices of insulin secretion, we used corrected insulin response (CIR=(100×insulin at 30 min)/((glucose at 30 min)×(glucose at 30 min–3.89))) and disposition index (DI=CIR×ISI).

### Statistical analyses

According to power analysis, 71 participants in each group were required to detect (power 80% and 95% CI) a 20% difference between the groups in achieving a 15% improvement in physical fitness. All data are reported as mean (SD or 95% CIs).

General linear models were used to compare the baseline characteristics between the groups (EG and PG stratified for FH). Linear mixed models were applied to test the differences between the PG and the EG during the 1-year and 5-year follow-up. The independent variables for these models were group (EG vs PG), time (baseline, 1 year, and 5 years), and FH and their interaction (group × time × FH). This model was employed to test the impact of FH on exercise responses (group × time × FH interaction effect) and the differences between the EG and PG (time × group interaction effect). Subjects who had data on all time-points for a specific variable were included in the analyses. Nominal p values are reported, and alpha level 0.05 was set as a threshold for statistical significance. Pearson correlation was used to measure correlation between the exercise dose during the 1-year intervention and the residual change in outcomes from baseline to 1 year.

The marginal mean weighting through stratification (MMWS) method was applied to estimate the average treatment effect between the intervention groups (PG and EG) and the CON group.^27–30^ MMWS incorporates two propensity score based techniques (stratification and weighting) to reweight the dataset. The propensity score for each treatment group was estimated with multinomial logistic regression, where treatment allocation (PG, EG, and CON) was explained by the pretreatment covariates (sex, age, BMI, follow-up time, and fitness index). Each of the three propensity scores was then stratified into tertiles to reduce the initial bias in the covariates.^31^ The generated weights were then incorporated in the regression model.

The 6-year response was compared between groups with and without FH and adjusted for baseline values. To correct for multiple comparisons (PG vs CON, EG vs CON, PG vs EG, and PG+EG vs CON), a p value <0.0125 (=0.05/4=0.0125) was used as a threshold for statistical significance in these analyses.

The generalized estimating equations were applied to compare the change in usage of medication for blood pressure and cholesterol between the CON, PG, and EG from baseline to 6-year follow-up. Penalized maximum likelihood logistic regression (Firth’s logistic regression) was used to compare the diabetes medication between the groups at 6 years, as none of the participants had diabetes medication at baseline, and the frequencies were low at 6 years.

All analyses were performed with Stata/SE V.14.2, Stata/MP V.15.1, and Stata/MP V.16.1.

## Results

### Baseline characteristics of the intervention groups

A total of 290 participants were randomized into the two intervention arms. Altogether 266 participants (136 in PG and 130 in EG), who completed the 5-year follow-up, were included in the primary analyses (online supplemental figure S2). Baseline characteristics between the intervention groups stratified for FH were similar expect for LDL-C (table 1). LDL-C was 0.4 mmol/L (95% CI 0.1 to 0.7) higher in the FH individuals in the exercise intervention compared with the FH individuals in the prescription group. No other significant differences were detected between the intervention groups.

**Table 1:** Baseline characteristics of individuals in the intervention groups stratified for family history of type 2 diabetes (FH)

Variable	Prescription group (PG)				Exercise group (EG)				P value
	FH− (n=71)		FH+ (n=73)		FH− (n=72)		FH+ (n=74)		
	n	Mean (SD)	n	Mean (SD)	n	Mean (SD)	n	Mean (SD)	
Males, n (%)	71	39 (55)	73	35 (48)	72	42 (58)	74	36 (48)	0.535
Age (years)	71	49.1 (12)	73	50.9 (10.6)	72	49.6 (11.8)	74	49.2 (10.6)	0.769
Weight (kg)	66	81.7 (13.2)	68	81.1 (14.8)	62	83.5 (14.5)	65	82.8 (15.3)	0.781
BMI (kg/m^2^)	71	27.5 (3.2)	71	28.2 (3.7)	72	28.2 (4.3)	74	28.1 (4.0)	0.643
BMI categories, n(%)									0.861
Normal weight (<25 kg/m^2^)	71	12 (17)	71	12 (17))	72	15 (21)	74	13 (18)	
Overweight (25–29.9 kg/m^2^)	71	45 (63)	71	39 (55)	72	40 (56)	74	40 (54)	
Obese (≥30 kg/m^2^)	71	14 (20)	71	20 (28)	72	17 (24)	74	21 (28)	
Waist circumference (cm)	66	95 (11.1)	68	93.4 (11.8)	62	97.2 (13.6)	65	95.3 (12.9)	0.379
Fitness index	71	73.8 (12.1)	73	73.3 (15.6)	72	72.2 (18.5)	74	73.1 (15)	0.932
LTPA (met-hours/week)	66	19 (20.2)	69	22.8 (22.7)	63	25.1 (22.8)	64	26 (19.9)	0.256
Number of steps	52	6.4 (2.5)	62	6.7 (2.5)	46	6.5 (2.4)	53	6.5 (2.9)	0.958
Blood pressure medication, n(%)	71	6 (13)	73	17 (23)	72	14 (19)	74	12 (16)	0.395
Lipid medication, n(%)	71	6 (8)	73	4 (5)	72	10 (14)	74	7 (9)	0.394
Systolic BP (mm Hg)	66	134.1 (15.3)	68	136.7 (14.5)	62	134.8 (15.4)	65	132.6 (14.4)	0.459
Diastolic BP (mm Hg)	71	82.6 (8.7)	73	81.4 (8.4)	72	82.7 (8.9)	74	82 (8.8)	0.804
Triglycerides (mmol/L)	71	1.23 (0.62)	73	1.27 (0.58)	72	1.37 (0.81)	74	1.39 (0.74)	0.437
Total cholesterol (mmol/L)	71	5.07 (0.88)	73	5.36 (0.9)	72	5.46 (0.9)	74	5.28 (1.06)	0.085
HDL cholesterol (mmol/L)	71	1.4 (0.43)	73	1.41 (0.41)	72	1.34 (0.39)	74	1.34 (0.38)	0.593
LDL cholesterol (mmol/L)	71	3.11 (0.8)	73	3.37 (0.85	69	3.51 (0.77)	72	3.31 (0.91)	0.039
Fasting glucose (mmol/L)	71	5.4 (0.5)	73	5.5 (0.6)	72	5.4 (0.5)	73	5.5 (0.5)	0.061
30 min glucose (mmol/L)	70	8.1 (1.6)	73	8.4 (1.5)	72	8 (1.5)	73	8.6 (1.7)	0.142
2-hour glucose (mmol/L)	71	5.2 (1.5)	73	5.4 (1.4)	72	5.1 (1.3)	73	5.4 (1.5)	0.641
AUC glucose	70	861.7 (139.1)	73	887.7 (127.2)	72	853.2 (119.9)	73	901 (145.7)	0.112
Fasting insulin (mU/L)	68	7.4 (7.3)	73	6.6 (3.7)	68	6.9 (4.5)	66	7.3 (4.7)	0.755
30 min insulin (mU/L)	67	63.4 (34.4)	72	61.5 (37.1)	68	65.4 (37)	66	70.6 (42.1)	0.529
2-hour insulin (mU/L)	68	32.4 (29.8)	70	31.1 (22.9)	68	31.4 (23.3)	65	32.3 (24.5)	0.986
AUC insulin (10^3^)	67	5.43 (2.94)	70	5.28 (2.92)	68	5.5 (3)	65	5.9 (3.16)	0.678
ISI	67	156.9 (87.8)	70	148 (69.5)	68	147.4 (76.9)	65	138.7 (86)	0.638
CIR	67	240.2 (289.8)	72	181.2 (111.5)	68	224.4 (171.1)	66	251.6 (356.1)	0.359
DI (10^3^)	67	34.2 (41.2)	70	25.7 (17.8)	68	31.5 (31.7)	65	29 (35.2)	0.474
HOMA-IR	69	1.8 (2.0)	71	1.6 (1.0)	67	1.7 (1.1)	66	1.8 (1.2)	0.750

Data are shown as mean (SD) unless otherwise stated.

AUC, area under the curve; BMI, body mass index; BP, blood pressure; CIR, corrected insulin response; DI, disposition index; FH, family history of type 2 diabetes; HDL, high-density lipoprotein; HOMA-IR, homeostasis model assessment for insulin resistance; ISI, insulin sensitivity index; LDL, low-density lipoprotein; LTPA, leisure time physical activity.

### Attendance for interventions and physical activity

During the 1-year intervention, 44% of the participants in the EG trained on average at least once and 11% trained at least twice per week. No significant differences in the dose of the intervention was observed between the FH− and FH+ groups (p>0.1) (online supplemental table S1). According to the exercise diaries, during the 1-year intervention, the EG performed on average more gym or resistance training type of exercise than the PG (online supplemental table S2). No significant differences were detected in aerobic type of exercises, miscellaneous physical activities or in total physical activity during the 1-year period. Also, we detected no significant between the group differences in volume of leisure time physical activity at baseline, 1 year or 5 years measured with 12-month KIHD recall questionnaire (online supplemental table S3).

### The influence of mode of intervention exercise response at 1 and 5 years

The EG had significantly lower body weight and waist circumference at 1 year, as well as lower total cholesterol, LDL-C, and diastolic BP (table 2) and better muscle strength (online supplemental table S3) at both 1 and 5 years compared with the PG (p≤0.019). Overall, however, the beneficial metabolic changes obtained at the 1-year follow-up in the intervention groups tended to fade at 5 years. No other significant differences were detected (online supplemental table S3). The sensitivity analyses for the main outcomes described in online supplemental table S6 indicated that these findings remained similar, even when we included all the participants in the analyses that had data on at least from one time-point (baseline, 1 year and 5 years). In addition, results remained similar when models were adjusted for baseline physical activity, physical activity by time interaction or volume of resistance training (data not shown). The correlation coefficients between the volume of exercise performed during the 1-year intervention and the residual change in outcomes from baseline to 1 year are described in the heat map in the online supplemental figure S3. There were no reported adverse effects in either group.

**Table 2:** The comparison of the effect of 1-year prescription-only intervention and supervised exercise intervention on physical fitness, body weight, waist circumference, blood pressure, lipid and glucose metabolism at 1 year and 5 years

Variable	N	Mean (SE)			Exercise versus prescription group change		P for time × group interaction
					Mean (95% CI)*		
		Baseline	1 year	5 years	1 year	5 years	
Fitness index							
Prescription	99	74.2 (1.2)	83.1 (83.1)	81.5 (1.6)			
Exercise	94	73.7 (1.4)	84.3 (84.3)	79.3 (1.8)	1.7 (−1.1 to 4.5)	−1.6 (−5.0 to 1.8)	0.074
Weight (kg)							
Prescription	134	81.4 (1.2)	81.1 (81.1)	82.0 (1.3)			
Exercise	127	83.2 (1.3)	81.9 (81.9)	84.2 (1.4)	−0.9 (−1.7 to −0.2)	0.4 (−0.7 to 1.5)	0.008
Waist circumference (cm)							
Prescription	134	94.2 (1.0)	92.3 (92.3)	94.9 (1.0)			
Exercise	127	96.2 (1.2)	93.2 (93.2)	97.4 (1.1)	−1.3 (−2.4 to −0.1)	0.5 (−0.9 to 1.8)	0.011
Systolic BP (mm Hg)							
Prescription	132	135.2 (1.3)	132.1 (132.1)	134.4 (1.4)			
Exercise	126	133.7 (1.3)	128.5 (128.5)	131.0 (1.4)	−2.1 (−4.7 to 0.5)	−1.9 (−4.9 to 1.2)	0.244
Diastolic BP (mm Hg)							
Prescription	132	81.9 (0.8)	78.8 (78.8)	76.0 (0.8)			
Exercise	126	82.6 (0.8)	77.9 (77.9)	74.2 (0.7)	−1.6 (−3.2 to −0.04)	−2.5 (−4.3 to −0.6)	0.019
Triglycerides (mmol/L)							
Prescription	136	1.27 (0.05)	1.27 (1.27)	1.32 (0.06)			
Exercise	128	1.4 (0.07)	1.25 (1.25)	1.44 (0.07)	−0.15 (−0.31 to 0.01)	−0.01 (−0.15 to 0.13)	0.147
Total cholesterol (mmol/L)							
Prescription	136	5.26 (0.08)	5.35 (5.35)	5.68 (0.09)			
Exercise	128	5.36 (0.09)	5.24 (5.24)	5.46 (0.09)	−0.21 (−0.41 to −0.01)	−0.32 (−0.52 to −0.12)	0.006
HDL-cholesterol (mmol/L)							
Prescription	136	1.40 (0.04)	1.47 (1.47)	1.42 (0.04)			
Exercise	128	1.35 (0.03)	1.41 (1.41)	1.32 (0.04)	−0.01 (−0.08 to 0.05)	−0.05 (−0.11 to 0.01)	0.210
LDL-cholesterol (mmol/L)							
Prescription	132	3.25 (0.07)	3.29 (3.29)	3.64 (0.08)			
Exercise	121	3.38 (0.08)	3.25 (3.25)	3.49 (0.08)	−0.17 (−0.35 to 0.004)	−0.28 (−0.46 to −0.10)	0.006
Fasting glucose (mmol/L)							
Prescription	135	5.4 (0.05)	5.3 (5.3)	5.5 (0.05)			
Exercise	129	5.5 (0.04)	5.4 (5.4)	5.6 (0.1)	0.0 (−0.1 to 0.1)	0.03 (−0.1 to 0.2)	0.831
30 min glucose (mmol/L)							
Prescription	131	8.3 (0.1)	8.2 (8.2)	8.5 (0.1)			
Exercise	123	8.4 (0.1)	8.3 (8.3)	8.8 (0.2)	0.03 (−0.3 to 0.4)	0.3 (−0.1 to 0.6)	0.307
2-hour glucose (mmol/L)							
Prescription	132	5.3 (0.1)	5.1 (5.1)	5.4 (0.1)			
Exercise	125	5.2 (0.1)	4.8 (4.8)	5.5 (0.1)	−0.2 (−0.6 to 0.1)	0.1 (−0.3 to 0.5)	0.123
Fasting insulin (mU/L)							
Prescription	130	7.1 (0.5)	6.1 (6.1)	7.3 (0.4)			
Exercise	126	7.2 (0.4)	6.7 (6.7)	8.4 (0.5)	0.4 (−0.7 to 1.6)	1 (−0.2 to 2.3)	0.231
30 min insulin (mU/L)							
Prescription	125	63.4 (3.2)	62.0 (62.0)	68.3 (3.8)			
Exercise	120	68.1 (3.7)	60.9 (60.9)	75.6 (4.9)	−5.8 (−13.4 to 1.8)	2.6 (−7.1 to 12.4)	0.202
2-hour insulin (mU/L)							
Prescription	124	32.3 (2.1)	27.9 (27.9)	36.2 (2.8)			
Exercise	117	31.6 (2.1)	25.6 (25.6)	37.2 (2.5)	−1.6 (−6.9 to 3.7)	1.8 (−4.1 to 7.6)	0.562
HOMA-IR							
Prescription	126	1.8 (0.1)	1.5 (1.5)	1.8 (0.1)			
Exercise	122	1.8 (0.1)	1.6 (1.6)	2.1 (0.1)	0.1 (−0.2 to 0.5)	0.3 (−0.1 to –0.6)	0.349

*Adjusted for family history of type 2 diabetes.

BP, blood pressure; HDL, high-density lipoprotein; HOMA-IR, homeostasis model assessment for insulin resistance; LDL, low-density lipoprotein.

### Exercise interventions versus population-based controls at 6 years

At 6 years (median=6.7 years, IQR=0.9), there were no significant differences between the PG and EG in change any of the outcomes (p>0.079) (online supplemental table S4), which is why we investigated contrast between the pooled intervention group (PG+EG) and the CON group. To compare effects of intervention with the status of the general population, we compared 275 randomized participants (PG+EG) and 782 matched control subjects (CON) who had participated in the 6-year prospective part of the PPP-Botnia Study (online supplemental figure S2).

The baseline characteristics were similar between the CON and the pooled intervention group, except for 0.1 mm Hg (95% CI 0.04 to 0.2) lower fasting glucose, 2.6 mmol/L (95% CI 1.3 to 4.0) lower diastolic blood pressure, and 8.1 IU/L (95% CI 2.3 to 13.8) higher 30 min insulin level in the pooled intervention compared with the CON group (table 3, online supplemental table S5).

Compared with CON group, the pooled intervention group had significantly lower systolic and diastolic blood pressure, insulin levels, HOMA-IR, and higher HDL-C at 6 years (table 3) and a nominally significant decrease in 2-hour glucose from the baseline. The intervention groups also showed a slightly higher increase in fasting glucose from baseline compared with the CON group, which could possibly be explained by the lower fasting glucose in the pooled intervention group at baseline (online supplemental table S5). The usage of blood pressure, cholesterol and diabetes medication increased similarly in each group during the 6-year period (online supplemental figure S4).

**Table 3:** The effect of pooled exercise interventions on body weight, waist circumference, blood pressure, lipid and glucose metabolism compared with the control group

Variable	Mean (SE)*				Pooled intervention versus CON change from baseline to 6 years†		P for group × FH intertaction term		
	Control group (CON) (n=782)		Pooled intervention group (n=275)						
	Baseline	6 years	Baseline	6 years	Mean (95% CI)*	P value	Group	FH	Interaction
Weight (kg)	82.0 (0.5)	83.2 (0.5)	82.4 (0.9)	83.0 (0.9)	−0.5 (−1.4 to 0.4)	0.272	0.464	0.148	0.147
Waist circumference (cm)	93.9 (0.4)	96.1 (0.4)	94.7 (0.7)	96.2 (0.7)	−0.7 (−1.5 to 0.2)	0.146	0.199	0.046	0.31
Body mass index (kg/m^2^)	28.0 (0.1)	28.4 (0.2)	28.0 (0.3)	28.3 (0.2)	−0.2 (−0.5 to 0.1)	0.322	0.585	0.189	0.176
Systolic BP (mm Hg)	134.8 (0.7)	139.7 (0.7)	133.4 (1.0)	133.9 (1.0)	−4.8 (−6.7 to −3.0)	<0.001	<0.001	0.412	0.218
Diastolic BP (mm Hg)	84.0 (0.4)	79.8 (0.4)	81.4 (0.6)	75.3 (0.6)	−2.9 (−4.0 to −1.9)	<0.001	<0.001	0.696	0.477
Triglycerides (mmol/L)	1.5 (0.03)	1.5 (0.03)	1.4 (0.05)	1.4 (0.05)	−0.1 (−0.2 to 0.01)	0.097	0.164	0.854	0.979
Total cholesterol (mmol/L)	5.5 (0.04)	5.5 (0.04)	5.5 (0.1)	5.6 (0.1)	0.1 (0.004 to 0.2)	0.042	0.029	0.992	0.07
HDL-cholesterol (mmol/L)	1.3 (0.01)	1.3 (0.01)	1.3 (0.02)	1.4 (0.03)	0.1 (0.03 to 0.1)	0.001	0.004	0.405	0.842
LDL-cholesterol (mmol/L)	3.5 (0.03)	3.5 (0.04)	3.6 (0.1)	3.6 (0.1)	0.1 (−0.02 to 0.2)	0.103	0.05	0.952	0.046
Fasting glucose (mmol/L)	5.4 (0.02)	5.5 (0.03)	5.2 (0.04)	5.6 (0.04)	0.2 (0.1 to 0.3)	<0.001	0.001	0.082	0.432
30 min glucose (mmol/L)	8.5 (0.1)	8.9 (0.1)	8.5 (0.1)	8.7 (0.1)	−0.2 (−0.4 to 0.01)	0.059	0.099	0.061	0.106
2-hour glucose (mmol/L)	5.3 (0.1)	5.9 (0.1)	5.2 (0.1)	5.5 (0.1)	−0.3 (−0.5 to −0.01)	0.038	0.066	0.398	0.962
AUC glucose	890.7 (4.96)	937.9 (7)	881 (8.7)	912.5 (10.5)	−19.1 (−39.1 to 0.9)	0.062	0.11	0.119	0.33
Fasting insulin (mmol/L)	7.7 (0.2)	9.2 (0.2)	7.9 (0.6)	7.8 (0.3)	−1.6 (−2.4 to −0.79)	<0.001	<0.001	0.008	0.591
30 min insulin (mmol/L)	63.6 (1.5)	76.5 (3)	70.8 (2.5)	73.5 (3.2)	−9.6 (−15.6 to −3.7)	0.002	0.003	0.869	0.443
2-hour insulin (mmol/L)	37.2 (1.4)	47.9 (2.1)	34.2 (1.9)	37.9 (2.0)	−8.4 (−13.5 to −3.3)	0.001	0.002	0.443	0.294
AUC insulin (10^3^)	5.7 (0.13)	6.96 (6.0)	6.0 (0.2)	6.23 (0.24)	−1.04 (−1.54 to −0.55)	<0.001	<0.001	0.51	0.321
ISI	142.4 (3.31)	120.3 (3)	139.1 (5)	134.5 (5.4)	16.9 (6.6 to 27.2)	0.001	0.003	0.057	0.841
CIR	198.2 (8.01)	208.9 (6.9)	241.8 (16.8)	207.7 (16.7)	−16.4 (−50.1 to 17.2)	0.337	0.623	0.187	0.056
DI (10^3^)	25.2 (1.16)	23.44 (1.12)	31.5 (2.78)	24.56 (3.13)	−0.17 (−6.36 to 6.03)	0.958	0.702	0.034	0.108
HOMA-IR	1.8 (0.05)	2.3 (0.1)	1.9 (0.2)	2.0 (0.1)	−0.4 (−0.6 to −0.1)	0.003	0.008	0.004	0.693

FH+=family history of diabetes.

*Propensity score weighted estimated means.

†Adjusted for baseline value and family history of diabetes.

AUC, area under the curve; BP, blood pressure; CIR, corrected insulin response; DI, disposition index; FH, family history of type 2 diabetes; HDL, high-density lipoprotein; HOMA-IR, homeostasis model assessment for insulin resistance; ISI, insulin sensitivity index; LDL, low-density lipoprotein.

### The influence of FH on exercise response

**Figure 1:**
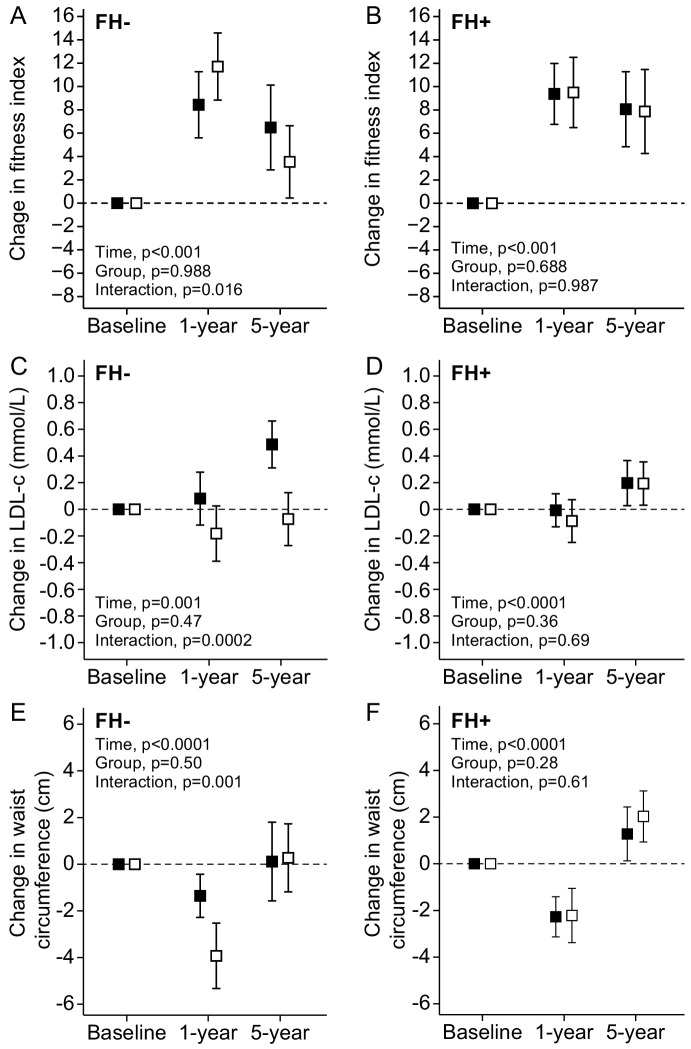
Mean change in fitness index (A and B), LDL-cholesterol (C and D) and waist circumference (E and F) in the prescription group (black square) and in the exercise intervention group (white square) in people with (FH+) or without (FH−) family history of diabetes. Error bars indicate 95% CI. FH, family history of type 2 diabetes; LDL, low-density lipoprotein.

FH modified the response of lipids and waist circumference to training. In FH− individuals, EG improved LDL-C at both 1 and 5 years and waist circumference at 1 year compared with the PG (figure 1C and E). No such effects were found in the FH+ group, which showed similar response to EG and PG interventions (figure 1D and F). At 1 year and 5 years, FH did not significantly influence the exercise response measured as aerobic fitness (p for time × group × FH interaction=0.11) (figure 1A and B) or any other outcomes.

At 6 years, there was a significant interaction between the mode of intervention (EG and PG) and FH on the change in LDL-C (group × FH interaction effect) (online supplemental figure S5). Among the FH− group, LDL-C decreased in the EG compared with the PG (p=0.023), but no such effect was detected among the FH+ group (p=0.815). However, among the FH+, both intervention groups had a similar increase in their LDL-C compared with the CON group, but this was statistically significant only for the PG (p=0.023). No other significant group × FH+ interactions were found (p>0.07). No other significant group × FH+ interactions were found (p>0.07) (table 3).

## Discussion

In an adult non-diabetic population with low cardiorespiratory fitness, we detected no difference in cardiorespiratory fitness between the 1 year EG or PG interventions at 1 or 5 years. The 1-year EG intervention, however, induced greater improvement in muscle strength, larger reduction in body weight and diastolic blood pressure, and LDL-cholesterol than the prescription only intervention. In addition, contrary to our hypothesis, we found no strong evidence that FH modifies individual responsiveness to exercise. Our findings suggest that individuals with FH may be less prone to reduce their LDL-C and possibly waist circumference with exercise, but overall, they achieve similar exercise-induced improvements in insulin resistance, HDL-C, and blood pressure as individuals without FH. The main finding was that irrespective of the mode of intervention, the 1-year low-cost exercise interventions resulted in a lower 6-year systolic and diastolic blood pressure, insulin levels, HOMA-IR and higher HDL-C compared with the background population, the CON group, taken from the same population study. Thus, the present findings support the conclusion that both EG and PG interventions have beneficial long-term effects on cardiometabolic health independent of the FH status.

The strength of the study is the population-based recruitment of the study participants and random allocation of participants into a training and prescription group. We also observed a very low overall dropout rate, as 91.7% of the participants participated in the 5-year examination. We were able to take a real-life control group from the PPP-Botnia cohort and to estimate the propensity score based average treatment effect of those who did or did not receive the intervention. Furthermore, participants reported no major adverse effects that can be traced back to the interventions. Thus, these interventions can be safely recommended for adult populations.

There are some limitations of the current study that should be addressed. As in many other studies, we encountered problems with monitoring physical activity and quantity and intensity of exercise interventions. The key instruments were 12-month recall questionnaire, the exercise diary and adherence to weekly exercise sessions, but this was left to the individual’s compliance. Although we aimed to test a less resource-consuming intervention, in future studies, it may be beneficial to include activating contacts, including new web-based technologies, by health professionals or trainers to increase the motivation and adherence to the intervention.

In the present study, individually given low-resource intensive exercise prescription intervention aiming for 150 min of exercise per week provided similar long-term cardiometabolic health benefits as the supervised exercise intervention in low-risk population. Participants that received either type of exercise intervention (PG+EG) improved their blood pressure, HDL-C, and insulin sensitivity, without a significant change in the waist circumference or body weight, compared with the CON group during the 6.8-year follow-up (table 3). Our findings support the findings from previous studies reporting beneficial cardiometabolic effects from unsupervised or counselling-based exercise interventions.^32–34^ These studies have, however, had relatively short follow-up time (6–12 months) and included mostly high-risk populations. According to the recent review including people with no cardiovascular disease risk factors, lifestyle interventions (diet and/or exercise) induced a small improvement in systolic and diastolic blood pressure, LDL-C, BMI, and waist circumference, but not in HDL-C.^8^ In the present study, we observed improvements in systolic and diastolic blood pressure, but not in LDL-C or in adiposity. However, exercise intervention groups improved HDL-C and insulin sensitivity compared with the CON group. The discrepancy between the studies is most likely explained by the type of intervention, longer follow-up, and inclusion of community-based control group rather than a randomly allocated control group in the present study. Despite small differences between the studies, the evidence strongly suggests that low-resource intensive exercise intervention is a feasible, low-cost alternative to promote long-term cardiometabolic health in low-risk individuals.

Both the PG and the EG performed similarly in most of the measured variables. The EG gained more muscle strength during the intervention, which can be explained by the fact that resistance training was the most common type of exercise (online supplemental table S1). Also, muscle strength measurements were only available from a subsample of the PG, which limits the generalizability of these findings.

FH did not influence aerobic fitness response to training. These findings are consistent with a previous study reporting similar increase in the VO_2max_ between the first-degree relatives of type 2 diabetic patients and controls after a 10-week aerobic exercise intervention.^15^ Previously, it has also been suggested, although informally tested, that FH+ group may require greater volume of exercise to achieve similar VO_2max_ gain as the FH− group.^16^ In the present study, such effects were unobserved, as the slope between the total volume (MET-hours) of exercise and the change in the fitness score were similar between the FH− and FH+ groups (p for total exercise volume × FH interaction=0.361, data not shown). The discrepancy between the findings is most likely explained by the differences in the dose (type, volume, and intensity) of the exercise interventions and that we only included low fit individuals and used indirect measure of physical fitness. Usage of walk test may have limited our ability to detect differences between the FH− and FH+ groups in fitness. However, in a large community-based study, direct measurement of VO_2max_ is infeasible. In fact, the walk-test based predicted fitness score correlates well with direct VO_2_ measurements, and it can be successfully applied in follow-up studies.^19^ Moreover, as both intervention groups improved their fitness score, the walk test was sensitive enough to capture intervention-induced changes in fitness. In addition, a previous study that used graded exercise test reported similar increases in fitness between the FH− and FH+ groups after 8 weeks of training.^35^ It should, however, be emphasized that the observed increase in fitness among FH+ does not associate with similar muscle adaptations (eg, insulin sensitivity) as in the FH− group.^15^

Previous studies have well established that regular exercise decreases waist circumference and reduces LDL-C level.^8^ In the present study, such benefits were only detected among the FH− group. It has previously been reported that FH reduces fat oxidation^36 37^ and high-fat diet induced fat oxidation.^38^ The reduced fat oxidation in response to high-fat diet was unexplained by the differences in VO_2max_,^38^ which could partly explain why FH influenced only on lipoprotein and fat metabolism, but not physical fitness. Taken together, we found no strong evidence supporting FH as a modifier of cardiometabolic response to exercise.

In conclusion, 1-year PG or EG intervention provide similar long-term cardiometabolic health benefits compared with the community-based control population. Although the FH modulated the LDL-C and waist circumference response to exercise, it seems that it does not systematically modify cardiometabolic response to exercise in any major way. These findings illustrate that low-resource promotion of physical activity in a clinical setting can be a feasible tool for enhancing long-term cardiometabolic health in an adult FH− or FH+ populations. We encourage healthcare policymakers and practitioners to take actions that promote incorporation of low-cost physical activity programs into the clinical practice.
