# Supplementary material for: Low-cost exercise interventions improve long-term cardiometabolic health independently of a family history of type 2 diabetes: a randomized parallel group trial
Source: BMJ Open Diabetes Res Care. 2020 Nov 20;8(2):e001377. doi: 10.1136/bmjdrc-2020-001377 (PMC7682194; doi:10.1136/bmjdrc-2020-001377)
Supplement: Supplementary data [file bmjdrc-2020-001377supp001.pdf]

Online-Only Supplemental Material

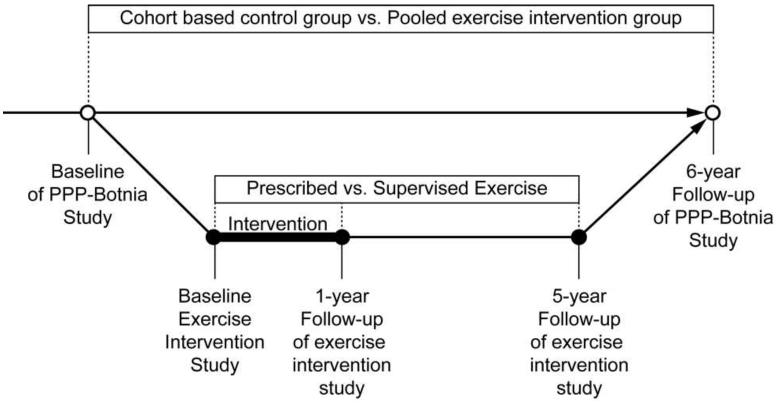

Supplemental Figure S1. The flow of the study

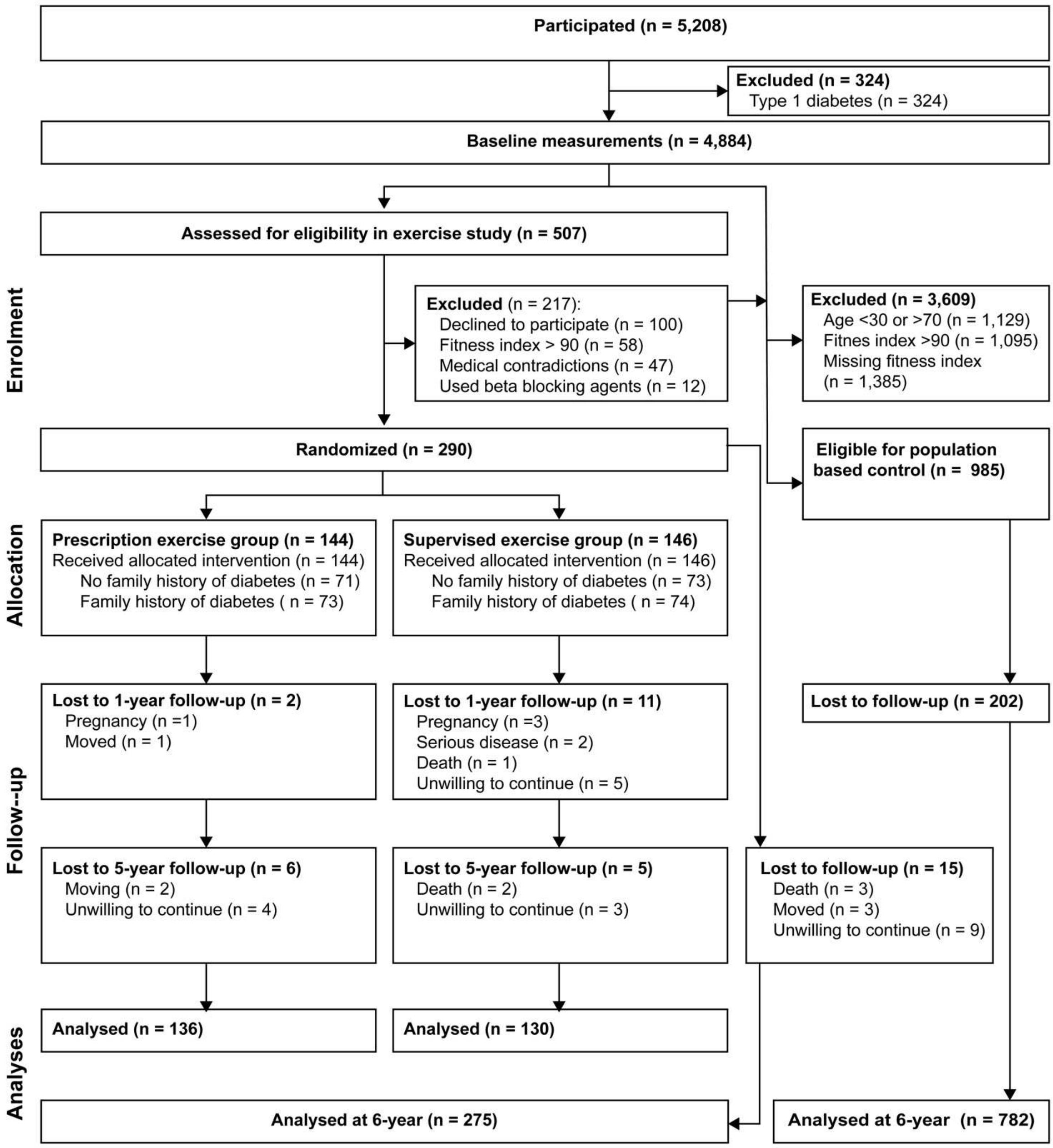

Supplemental Figure S2. Flow chart of participants.

Supplemental Table S1. Physical dose of the 12-month supervised exercise intervention

Variable	Median (IQR)	
Number of sessions (n/12 months)		
Nordic walking	4	(18)
Resistance training	30	(38)
Aquatic training	0	(10)
Total	46	(58)
Duration (min/12 months)		
Nordic walking	240	(1080)
Resistance training	1800	(2280)
Aquatic training	0	(600)
Total	2760	(3480)
Intensity (MET)		
Time-weighted average intensity (MET)	4	(0.5)
Volume (MET-hours/12 months)		
Nordic walking	19.2	(86.4)
Resistance training	105	(133)
Aquatic training	0	(55)
Total	182.3	(235)

MET = Metabolic equivalent of task, IQR = interquartile range

Supplemental Table S2. Comparison of volume of physical activity between prescription and exercise group during a 1-year intervention.

Variable	Prescription group (PG), n=142	Exercise group (EG), n=135	Between group comparison	
	**Mean (SD)**	**Mean (SD)**	**Mean difference (95% CI)**	**P**
Aerobic exercises (METh/wk)	13.2 (10.6)	13.9 (9.8)	0.8 (-1.7 – 3.1)	0.54
Resistance exercises (METh/wk)	0.9 (2.2)	3.6 (2.9)	2.6 (-2.0 – 3.2)	<0.001
Miscellaneous physical activity (METh/wk)*	8.1 (16.5)	9.6 (18.7)	1.5 (-2.5 – 5.8)	0.49
Total physical activity (METh/wk)	22.2 (19.6)	27.1 (22.0)	4.9 (-0.3 – 9.7)	0.057

*includes e.g. home activities, home repair, lawn and gardening, self-care

Supplemental Table S3. The effect of 1-year supervised exercise intervention on physical activity, muscle strength, and insulin and glucose metabolism at 1-year and 5-years.

Variable	n	Mean (SE)			Exercise vs Prescription group change, Mean (95% CI)		P for time x group interaction
		Baseline	1-year	5-years	1-year	5-years	
PA (METh/wk)							
Prescription	135	20.9 (1.8)	23.8 (23.8)	19.9 (1.5)			
Exercise	127	25.6 (1.9)	29.7 (29.7)	25.6 (1.9)	1.3 (-4.4 – 7.0)	1.1 (-4.7 – 6.8)	0.883
PA (min/wk)							
Prescription	135	199.4 (17.5)	226.6 (20.7)	189.7 (14.6)			
Exercise	127	243.4 (18.0)	282.6 (21.4)	244.2 ((17.8)	12.1 (-42.3 – 66.6)	10.3 (-44.3 – 64.9)	0.883
1000 Steps/d							
Prescription	114	6.5 (0.2)	7.3 (7.3)	6.4 (0.2)			
Exercise	99	6.5 (0.3)	7.7 (7.7)	6.8 (0.3)	0.4 (-0.3 – 1.1)	0.4 (-0.4 – 1.1)	0.488
Arm push (kg)							
Prescription	24	27.0 (2.2)	27.0 (27.0)	28.9 (2.5)			
Exercise	86	27.1 (1.5)	33.4 (33.4)	31.7 (1.8)	6.3 (4.0 – 8.5)	2.6 (0.3 – 4.9)	<0.001
Arm pull (kg)							
Prescription	22	44.8 (3.1)	45.2 (45.2)	50.8 (3.9)			
Exercise	86	44.0 (1.6)	52.9 (52.9)	54.4 (1.9)	8.6 (5.3 – 11.9)	4.3 (-1.2 – 9.9)	<0.001
Biceps curl (kg)							
Prescription	15	13.5 (1.5)	14.2 (14.2)	14.3 (1.6)			
Exercise	43	16.0 (2.1)	22.6 (22.6)	20.6 (3.0)	5.8 (3.1 – 8.4)	3.1 (0.5 – 5.7)	<0.001
Triceps extension (kg)							
Prescription	14	18.5 (1.3)	19.9 (19.9)	21.5 (1.9)			
Exercise	42	23.1 (2.9)	33.2 (33.2)	31.2 (3.7)	9.1 (6.0 – 12.1)	5.4 (2.0 – 8.7)	<0.001
Leg extension (kg)							
Prescription	22	21.2 (1.6)	24.2 (24.2)	24.2 (2.1)			
Exercise	87	24.6 (1.6)	31.5 (31.5)	30.4 (1.8)	4.0 (1.6 – 6.4)	2.7 (0.6 – 4.9)	0.002
Leg curl (kg)							
Prescription	24	24.6 (1.5)	26 (26)	26.9 (1.6)			
Exercise	88	27.9 (1.3)	35 (35)	34.6 (1.8)	5.8 (3.8 – 7.7)	4.4 (2.5 – 6.3)	<0.001

AUC glucose							
Prescription	130	816.0 (11.6)	805.1 (805.1)	836.3 (13.5)			
Exercise	123	817.5 (11.6)	796.7 (796.7)	859.4 (14.6)	-9.8 (-37.9 – 18.2)	21.7 (-8.9 – 52.3)	0.109
AUC insulin (10^3)							
Prescription	121	5.43 (0.27)	5.10 (0.26)	5.86 (0.34)			
Exercise	115	5.60 (0.27)	4.93 (0.24)	6.20 (0.32)	-0.34 (-0.93 – 0.25)	0.16 (-0.54 – 0.86)	0.338
ISI							
Prescription	120	149.7 (6.8)	168.3 (7.7)	146.5 (8.5)			
Exercise	114	144.6 (7.9)	165.8 (8.1)	131.9 (8.1)	2.5 (-13.5 – 0.02)	-9.4 (-27.6 – 0.01)	0.430
CIR							
Prescription	124	210.5 (20.2)	215.8 (16.9)	196.8 (29.9)			
Exercise	119	240.3 (26.9)	212.1 (17.1)	204.2 (14.6)	-33.5 (-83.6 – 16.7)	-22.2 (-104.9 – [illegible]	0.417
DI (10^3)							
Prescription	120	29.67 (2.99)	34.51 (3.42)	23.20 (6.41)			
Exercise	114	28.37 (2.81)	31.91 (3.45)	24.51 (2.17)	-1.31 (-10.18 – 7.57)	2.68 (-11.63 – 17)	0.883

Models adjusted for family history of type 2 diabetes

Supplemental Table S4. Baseline characteristics between the pooled intervention group and the population-based control group

	Control group	Pooled intervention group
**Baseline characteristics**	**Mean (SE)**	**Mean (SE)**
Female, n(%)	324 (41)	130 (47)
FH+, n(%)	296 (38)	140 (51)
Age*	50.8 (0.4)	49.8 (0.7)
Fitness index*	71.6 (0.6)	70.8 (1.2)
LTPA (MET-hours/wk)*	22.4 (1.2)	23.0 (1.7)
Follow-up time*	6.8 (0.02)	6.8 (0.1)

*Propensity score weighted estimated means

FH+ = family history of diabetes, LTPA = leisure-time PA

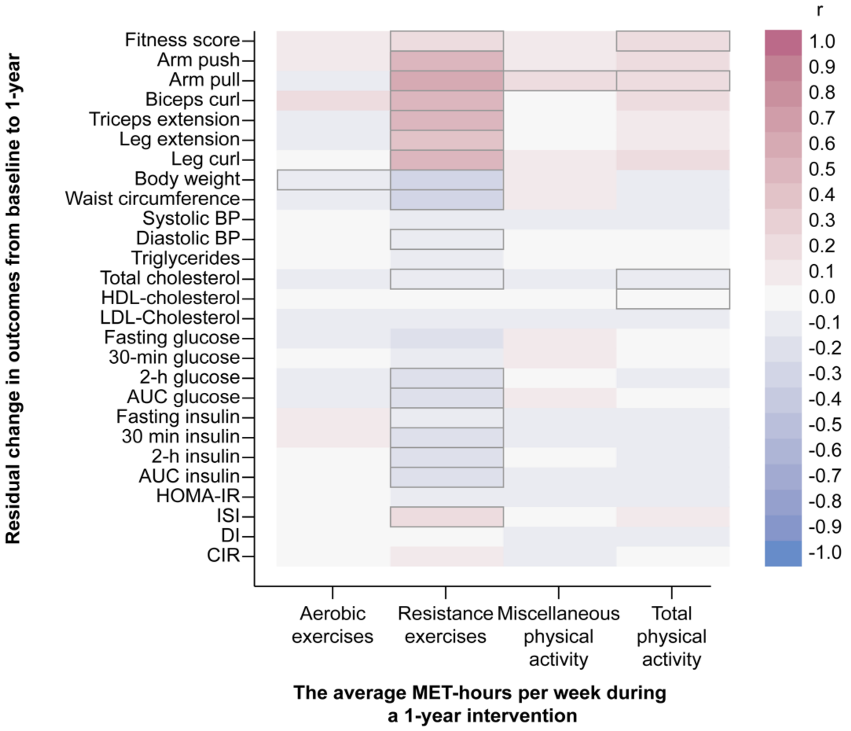

Supplemental Figure S3. Correlation between the residual change in outcomes from baseline to 1-year and the volume of physical exercise performed during the intervention. BP = blood pressure, HDL = high-density lipoprotein, LDL = low-density lipoprotein, AUC = area under the curve, HOMA = homeostatic model assessment for insulin resistance, ISI = insulin sensitivity index, DI= disposition index, CIR = corrected insulin response, MET = metabolic equivalent of task. Grey squares indicate the nominal statistical significance ($p<0.05$).

Supplemental Table S5. The effect of prescribed and supervised exercise interventions on body weight, waist circumference, blood pressure, lipid and glucose metabolism compared to the control group.

	**Mean (SE)***		**Between the group difference in change from baseline to 6-years****	
**Variable**	**Baseline**	**6-years**	**Mean (95% CI)**	**P-value**
PA (METh/wk)				
Control	22.3 (1.2)			
Prescription	24.2 (2.4)			
Exercise	22.1 (2.5)			
Precsiption vs control†			1.9 (-3.4 – 7.2)	0.489
Exercise vs control†			-0.2 (-5.7 – 5.2)	0.929
Exercise vs prescription†			-2.1 (-8.8 – 4.6)	0.534
Weight (kg)				
Control	82.0 (0.5)	83.2 (0.5)		
Prescription	82.2 (1.4)	82.6 (1.3)		
Exercise	82.5 (1.2)	83.5 (1.3)		
Precsiption vs control			-0.8 (-2.1 – 0.5)	0.218
Exercise vs control			-0.2 (-1.2 – 0.8)	0.712
Exercise vs prescription			0.6 (-0.8 – 2.1)	0.406
Waist circumference (cm)				
Control	93.9 (0.4)	96.1 (0.4)		
Prescription	94.5 (1.1)	95.5 (1.0)		
Exercise	94.9 (0.9)	96.8 (1.0)		
Precsiption vs control			-1.1 (-2.4 – 0.1)	0.072
Exercise vs control			-0.2 (-1.2 – 0.9)	0.746
Exercise vs prescription			1.0 (-0.5 – 2.4)	0.184
Body mass index (kg/m2)				
Control	28.0 (0.1)	28.4 (0.2)		
Prescription	28.0 (0.4)	28.2 (0.3)		
Exercise	28.0 (0.3)	28.3 (0.4)		
Precsiption vs control			-0.2 (-0.7 – 0.2)	0.318
Exercise vs control			-0.1 (-0.4 – 0.2)	0.626
Exercise vs prescription			0.1 (-0.3 – 0.6)	0.565
Systolic BP (mmHg)				
Control	134.8 (0.7)	139.7 (0.7)		
Prescription	134.8 (1.4)	135.9 (1.4)		
Exercise	132.1 (1.3)	131.8 (1.4)		
Precsiption vs control			-3.7 (-6 – -1.4)	0.002
Exercise vs control			-6.0 (-8.4 – -3.5)	<0.001
Exercise vs prescription			-2.3 (-5.2 – 0.7)	0.135
Diastolic BP (mmHg)				
Control	84.0 (0.4)	79.8 (0.4)		
Prescription	82.3 (0.9)	76.2 (0.8)		
Exercise	80.4 (0.7)	74.4 (0.8)		
Precsiption vs control			-2.6 (-3.9 – -1.2)	<0.001
Exercise vs control			-3.3 (-4.7 – -1.9)	<0.001
Exercise vs prescription			-0.7 (-2.4 – 1)	0.392

Triglycerides (mmol/L)				
Control	1.5 (0.03)	1.5 (0.03)		
Prescription	1.4 (0.1)	1.3 (0.1)		
Exercise	1.4 (0.1)	1.4 (0.1)		
Precsiption vs control			-0.1 (-0.2 – 0.01)	0.061
Exercise vs control			-0.03 (-0.1 – 0.1)	0.556
Exercise vs prescription			0.1 (-0.1 – 0.2)	0.292
Total cholesterol (mmol/L)				
Control	5.5 (0.04)	5.5 (0.04)		
Prescription	5.6 (0.1)	5.7 (0.1)		
Exercise	5.5 (0.1)	5.5 (0.1)		
Precsiption vs control			0.2 (0.1 – 0.4)	0.008
Exercise vs control			0.03 (-0.1 – 0.2)	0.661
Exercise vs prescription			-0.2 (-0.4 – 0.02)	0.079
HDL cholesterol (mmol/L)				
Control	1.3 (0.01)	1.3 (0.01)		
Prescription	1.4 (0.03)	1.4 (0.04)		
Exercise	1.3 (0.03)	1.3 (0.04)		
Precsiption vs control			0.1 (0.03 – 0.1)	0.003
Exercise vs control			0.05 (-0.003 – 0.1)	0.064
Exercise vs prescription			-0.04 (-0.1 – 0.04)	0.333
LDL cholesterol (mmol/L)				
Control	3.5 (0.03)	3.5 (0.04)		
Prescription	3.6 (0.1)	3.7 (0.1)		
Exercise	3.6 (0.1)	3.5 (0.1)		
Precsiption vs control			0.2 (0.03 – 0.3)	0.015
Exercise vs control			0.01 (-0.1 – 0.2)	0.889
Exercise vs prescription			-0.2 (-0.3 – 0.02)	0.079
Fasting glucose (mmol/L)				
Control	5.4 (0.02)	5.5 (0.03)		
Prescription	5.2 (0.05)	5.5 (0.05)		
Exercise	5.3 (0.1)	5.6 (0.1)		
Precsiption vs control			0.2 (0.1 – 0.3)	0.001
Exercise vs control			0.2 (0.1 – 0.3)	0.003
Exercise vs prescription			0.002 (-0.1 – 0.1)	0.976
30-min glucose (mmol/L)				
Control	8.5 (0.1)	8.9 (0.1)		
Prescription	8.4 (0.1)	8.6 (0.1)		
Exercise	8.5 (0.2)	8.8 (0.2)		
Precsiption vs control			-0.3 (-0.5 – -0.02)	0.037
Exercise vs control			-0.1 (-0.4 – 0.2)	0.372
Exercise vs prescription			0.1 (-0.2 – 0.5)	0.436
2-h glucose (mmol/L)				
Control	5.3 (0.1)	5.9 (0.1)		
Prescription	5.3 (0.1)	5.5 (0.1)		
Exercise	5.2 (0.1)	5.6 (0.2)		
Precsiption vs control			-0.3 (-0.6 – -0.04)	0.025
Exercise vs control			-0.2 (-0.5 – 0.2)	0.265
Exercise vs prescription			0.1 (-0.3 – 0.5)	0.511
AUC glucose				
Control	890.7 (4.96)	937.9 (7.0)		
Prescription	877.7 (12.4)	903.6 (13.1)		
Exercise	884.3 (12.3)	921.3 (16.2)		
Interventions	881.0 (8.7)	912.5 (10.5)		

Precsiption vs control			-25.5 (-50.0 – -1.0)	0.041
Exercise vs control			-12.7 (-39.6 – 14.3)	0.355
Exercise vs prescription			12.8 (-19.6 – 45.1)	0.438
Fasting insulin (mmol/L)				
Control	7.7 (0.2)	9.2 (0.2)		
Prescription	6.6 (0.3)	7.4 (0.4)		
Exercise	9.1 (1.2)	8.3 (0.5)		
Precsiption vs control			-1.6 (-2.4 – -0.8)	<0.001
Exercise vs control			-1.6 (-2.7 – -0.5)	0.005
Exercise vs prescription			0.03 (-1.1 – 1.2)	0.957
30-min insulin (mmol/L)				
Control	63.6 (1.5)	76.5 (2.0)		
Prescription	68.8 (3.1)	69.6 (3.9)		
Exercise	72.8 (4.0)	77.4 (5.0)		
Precsiption vs control			-11.9 (-19.1 – -4.6)	0.001
Exercise vs control			-7.4 (-15.8 – 1.0)	0.082
Exercise vs prescription			4.4 (-5.7 – 14.5)	0.390
2-h insulin (mmol/L)				
Control	37.2 (1.4)	47.9 (2.1)		
Prescription	35.8 (2.7)	37.1 (2.8)		
Exercise	32.6 (2.7)	38.7 (2.9)		
Precsiption vs control			-10.6 (-16.7 – -4.4)	0.001
Exercise vs control			-6.2 (-12.9 – 0.5)	0.070
Exercise vs prescription			4.4 (-3.5 – 12.2)	0.275
AUC insulin				
Control	5.7 (0.13)	6.96 (0.19)		
Prescription	5.9 (0.27)	6.04 (0.33)		
Exercise	6.0 (0.3)	6.42 (0.35)		
Interventions	6.0 (0.2)	6.23 (0.24)		
Precsiption vs control			-1.2 (-1.83 – -0.57)	<0.001
Exercise vs control			-0.88 (-1.51 – -0.26)	0.006
Exercise vs prescription			0.32 (-0.46 – 1.09)	0.427
ISI				
Control	25.2 (1.16)	23.44 (1.12)		
Prescription	33.4 (4.63)	22.91 (5.65)		
Exercise	29.5 (2.98)	26.27 (2.47)		
Interventions	31.5 (2.78)	24.56 (3.13)		
Precsiption vs control			22.0 (8.5 – 35.5)	0.001
Exercise vs control			11.8 (-2.1 – 25.7)	0.097
Exercise vs prescription			-10.3 (-28.4 – 7.9)	0.268
CIR				
Control	142.4 (3.31)	120.3 (3.0)		
Prescription	140.2 (5.5)	139.8 (7.6)		
Exercise	138.0 (8.5)	128.9 (7.5)		
Interventions	139.1 (5.0)	134.5 (5.4)		
Precsiption vs control			-25.6 (-81.4 – 30.2)	0.369
Exercise vs control			-7.3 (-42.5 – 27.9)	0.683
Exercise vs prescription			18.3 (-46.4 – 83.0)	0.580
DI				
Control	198.2 (8.01)	208.9 (6.9)		
Prescription	241.0 (26.1)	196.7 (27.4)		
Exercise	242.5 (20.9)	219.0 (18.8)		
Interventions	241.8 (16.8)	207.7 (16.7)		
Precsiption vs control			-2.11 (-13.07 – 8.86)	0.706

Exercise vs control			1.77 (-3.09 – 6.64)	0.474
Exercise vs prescription			3.88 (-7.71 – 15.47)	0.511
HOMA-IR				
Control	1.8 (0.05)	2.3 (0.1)		
Prescription	1.5 (0.1)	1.8 (0.1)		
Exercise	2.2 (0.3)	2.1 (0.1)		
Precsiption vs control			-0.4 (-0.6 – -0.1)	0.003
Exercise vs control			-0.4 (-0.7 – -0.03)	0.031
Exercise vs prescription			0.01 (-0.3 – 0.3)	0.96

*Propensity score weighted estimated means, **Adjusted for baseline value and family history of diabetes, †Comparison of between group difference at baseline only (propensity score weighted estimated means and adjusted for family history of diabetes)

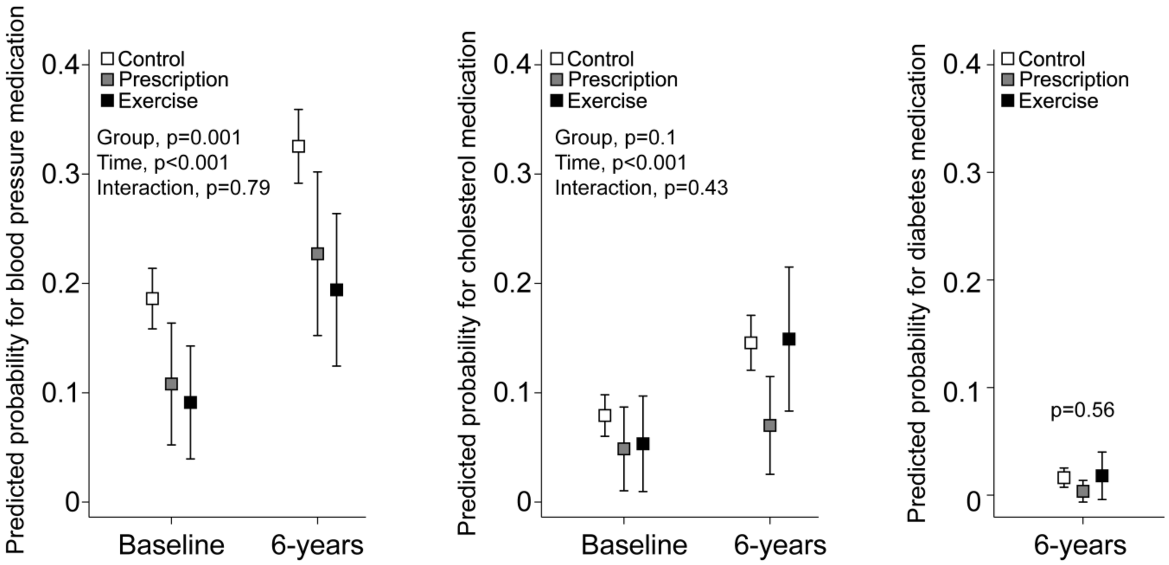

Supplemental Figure S4. Comparison of usage of blood pressure, cholesterol and diabetes medication between the control group, the prescription group and the supervised exercise group from baseline to 6-year follow-up. For usage of diabetes medication was compared only at 6-years as none of the participants used diabetes medication at baseline. The error bars indicate the 95% confidence intervals.

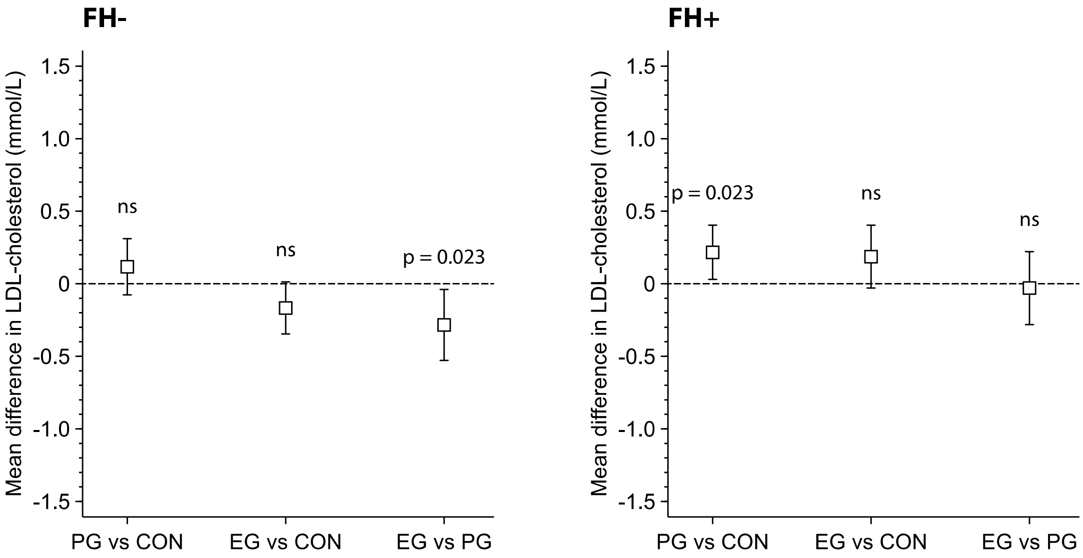

Supplemental Figure S5. The influence of FH on exercise response on low-density lipoprotein (LDL) cholesterol during the 6-year follow-up. PG = prescription group, EG = Supervised exercise intervention group, CON = matched control group from the same population study, FH = family history. Error bars indicate 95% CI.

BMJ Publishing Group Limited (BMJ) disclaims all liability and responsibility arising from any reliance placed on this supplemental material which has been supplied by the author(s)

Supplemental Table S6. Sensitivity analyses of main outcomes of the effect of supervised exercise intervention compared to the prescription group in participant with data for particular variable from at least one time point (baseline, 1-year or 5- years) in respect to the analyses including participants with data on each timepoint.

	Participants who had data at least from one timepoint				Participants who had data on each timepoint				
Variable	n	Mean between group difference (95% CI)*	P for time x group	P for time x group x FH	n	Mean between group difference (95% CI)*	P for time x group	P for time x group x FH	Percentage difference in sample sizes
Fitness index									
1 year vs baseline	290	2.9 (0.4 – 5.3)	0.008	0.434	193	1.7 (-1.1 – 4.5)	0.074	0.118	33.4
5 year vs baseline		-1.2 (-4.3 – 2)				-1.6 (-5 – 1.8)			
Weight (kg)									
1 year vs baseline	290	-1 (-1.7 – -0.2)	0.005	0.125	261	-0.9 (-1.7 – -0.2)	0.008	0.141	10.0
5 year vs baseline		0.4 (-0.7 – 1.5)				0.4 (-0.7 – 1.5)			
Waist circumference (cm)									
1 year vs baseline	290	-1.3 (-2.4 – -0.2)	0.007	0.046	261	-1.3 (-2.4 – -0.1)	0.011	0.047	10.0
5 year vs baseline		0.5 (-0.9 – 1.8)				0.5 (-0.9 – 1.8)			
Systolic BP (mmHg)									
1 year vs baseline	290	-2.1 (-4.6 – 0.5)	0.217	0.383	258	-2.1 (-4.7 – 0.5)	0.244	0.388	11.0
5 year vs baseline		-1.9 (-5 – 1.1)				-1.9 (-4.9 – 1.2)			
Diastolic BP (mmHg)									
1 year vs baseline	290	-1.3 (-2.9 – 0.2)	0.056	0.787	258	-1.6 (-3.2 – 0)	0.019	0.977	11.0
5 year vs baseline		-2.1 (-3.9 – -0.3)				-2.5 (-4.3 – -0.6)			
Triglycerides (mmol/L)									
1 year vs baseline	290	-0.14 (-0.29 – 0.01)	0.177	0.561	264	-0.2 (-0.3 – 0.01)	0.147	0.608	9.0
5 year vs baseline		-0.02 (-0.2 – 0.1)				-0.01 (-0.1 – 0.1)			
Total cholesterol (mmol/L)									

1 year vs baseline	290	-0.2 (-0.4 – -0.03)	0.001	0.031	264	-0.2 (-0.4 – -0.01)	0.006	0.024	9.0
5 year vs baseline		-0.4 (-0.6 – -0.2)				-0.3 (-0.5 – -0.1)			
HDL cholesterol (mmol/L)									
1 year vs baseline	290	-0.01 (-0.1 – 0.1)	0.226	0.214	264	-0.01 (-0.1 – 0.1)	0.21	0.175	9.0
5 year vs baseline		-0.1 (-0.1 – 0)				-0.1 (-0.1 – 0.01)			
LDL cholesterol (mmol/L)									
1 year vs baseline	290	-0.2 (-0.3 – 0.01)	0.001	0.009	253	-0.2 (-0.3 – 0.004)	0.006	0.008	12.8
5 year vs baseline		-0.3 (-0.5 – -0.1)				-0.3 (-0.5 – -0.1)			
Fasting glucose (mmol/L)									
1 year vs baseline	290	0 (-0.1 – 0.1)	0.888	0.604	264	0 (-0.1 – 0.1)	0.831	0.551	9.0
5 year vs baseline		0 (-0.1 – 0.2)				0.04 (-0.1 – 0.2)			
30-min glucose (mmol/L)									
1 year vs baseline	290	0.1 (-0.2 – 0.4)	0.463	0.224	254	0.03 (-0.3 – 0.4)	0.307	0.236	12.4
5 year vs baseline		0.2 (-0.1 – 0.6)				0.3 (-0.1 – 0.6)			
2-hour glucose (mmol/L)									
1 year vs baseline	290	-0.2 (-0.5 – 0.2)	0.253	0.944	257	-0.2 (-0.6 – 0.1)	0.123	0.932	11.4
5 year vs baseline		0.2 (-0.2 – 0.5)				0.1 (-0.3 – 0.5)			
Fasting insulin (mU/L)									
1 year vs baseline	286	0.4 (-0.8 – 1.5)	0.23	0.537	256	0.4 (-0.7 – 1.6)	0.231	0.665	10.5
5 year vs baseline		1 (-0.2 – 2.2)				1 (-0.2 – 2.3)			
30-min insulin (mU/L)									
1 year vs baseline	286	-3.8 (-11.1 – 3.4)	0.335	0.757	245	-5.8 (-13.4 – 1.8)	0.202	0.945	14.3
5 year vs baseline		3.5 (-5.9 – 12.8)				2.6 (-7.1 – 12.4)			
2-hour insulin (mU/L)									
1 year vs baseline	285	-1.8 (-7 – 3.4)	0.378	0.249	241	-1.6 (-6.9 – 3.7)	0.562	0.41	15.4
5 year vs baseline		2.5 (-3.5 – 8.4)				1.8 (-4.1 – 7.6)			
HOMA-IR									
1 year vs baseline	283	0.1 (-0.2 – 0.4)	0.245	0.622	248	0.1 (-0.2 – 0.5)	0.349	0.799	12.4
5 year vs baseline		0.3 (-0.1 – 0.6)				0.3 (-0.1 – 0.6)			

All analyses are based on the similar linear mixed model.
* The mean difference describes how much the supervised exercise group deviates from the prescription group.
